# Fractional Flow Reserve Implications for Clinical Decision Making in Coronary Artery Disease

**DOI:** 10.3390/life14101326

**Published:** 2024-10-18

**Authors:** Andrei Grib, Marcel Abras, Artiom Surev, Livi Grib

**Affiliations:** 1 Discipline of Cardiology, State University of Medicine and Pharmacy “Nicolae Testemitanu”, MD 2004 Chisinau, Moldovaltgrib@yahoo.com (L.G.); 2 Institute of Cardiology, MD 2025 Chisinau, Moldova

**Keywords:** fractional flow reserve, coronary artery disease, visual assessment variability, clinical decision making

## Abstract

Fractional flow reserve (FFR) is regarded as the gold standard for assessing the functional significance of coronary artery lesions. However, its utilization in clinical practice remains limited. This study aims to determine whether FFR results can influence treatment decisions for coronary artery disease compared to visual assessments of angiographic images. We conducted a retrospective study involving 63 patients diagnosed with either chronic coronary syndrome (*n* = 39, 61.9%) or acute coronary syndrome (*n* = 24, 38.1%) who underwent an FFR assessment. Three experienced interventional cardiologists (>300 PCI procedures/year) reevaluated 105 ambiguous coronary lesions in these patients, blinded to the FFR results. The objective was to assess lesion significance and determine the treatment strategy based on a visual angiographic evaluation. The three operators reached concordant agreement (≥two operators) to perform PCI in 60 (57.1%) of the evaluated lesions based on the angiographic assessment. Of these, nine lesions (15%) were deemed functionally non-significant by FFR (FFR > 0.80). Conversely, they agreed to defer PCI in 45 (42.9%) lesions, but 4 lesions (8.9%) were found to be functionally significant (FFR ≤ 0.80) and required a re-evaluation for PCI. Visual-guided decision making by interventional cardiologists shows variability and does not always align with the functional significance of coronary lesions as determined by FFR. Incorporating FFR into routine decision making could enhance treatment accuracy and patient outcomes.

## 1. Introduction

The precise evaluation of myocardial ischemia is crucial in the management of coronary artery disease (CAD) for making informed decisions about coronary revascularization [1]. Fractional flow reserve (FFR) is a pressure-derived estimate of coronary flow impairment and one of the most established invasive physiological tests in interventional cardiology. It is defined as the ratio between the mean hyperemic coronary artery pressure distal to the lesion and mean pressure in the aorta. FFR is an invasive index of the hemodynamic significance of stenosis severity with a diagnostic accuracy similar to myocardial perfusion scintigraphy [2]. European guidelines recommend performing fractional flow reserve (FFR) measurements prior to revascularization, particularly when previous ischemia testing is inconclusive or absent [3]. However, despite the growing preference for physiologically guided revascularization, there remains a significant gap between guideline recommendations and the actual clinical use of the functional assessment of the lesions [4,5].

Hemodynamic significance, as defined by an FFR ≤ 0.80, shows a weak correlation with the visually assessed diameter of the stenosis. In the FAME trial (Fractional Flow Reserve versus Angiography for Guidance of PCI in Patients with Multivessel Coronary Artery Disease), only 35% of stenoses assessed by invasive quantitative coronary angiography (CA) in the 50–70% range were hemodynamically significant, and among stenoses in the 71–90% range, 20% were not significant. Only a stenosis diameter estimated at >90% was found to be hemodynamically significant, with a high accuracy of 96% [6]. CA is the current gold-standard method of CAD assessment, but it fails to adequately determine lesion significance because lumen narrowing is just one of many variables influencing coronary flow limitation. Other critical factors that are not easily quantifiable by CA include lesion length, collateral flow, and the amount and viability of the myocardium supplied downstream [7,8]. Several studies have shown that an FFR-based evaluation at the time of CA leads to the reclassification of revascularization strategies (PCI, coronary artery bypass grafting, or medical treatment) in a significant proportion of patients with intermediate-grade lesions (>40% of patients were reclassified) [9,10,11]. However, the impact of FFR on determining revascularization tactics may be of greater interest for patients with multivessel CAD, giving us the opportunity to minimally intervene on functionally significant lesions and safely defer insignificant ones [12].

Unlike FFR, which is an objective method for assessing lesions capable of inducing ischemia, CA is a subjective method for evaluating the degree of arterial lumen stenosis, dependent on the observer. The inter-observer agreement in visually guided therapeutic decision making is variable [13] and not always confirmed by the functional significance of coronary lesions [14]. Given this variability, our study aimed to evaluate the differences in the visual assessment of CAD by experienced operators and to assess the impact of FFR on clinical decision making.

## 2. Materials and Methods

### 2.1. Patient Selection

**Figure 1:**
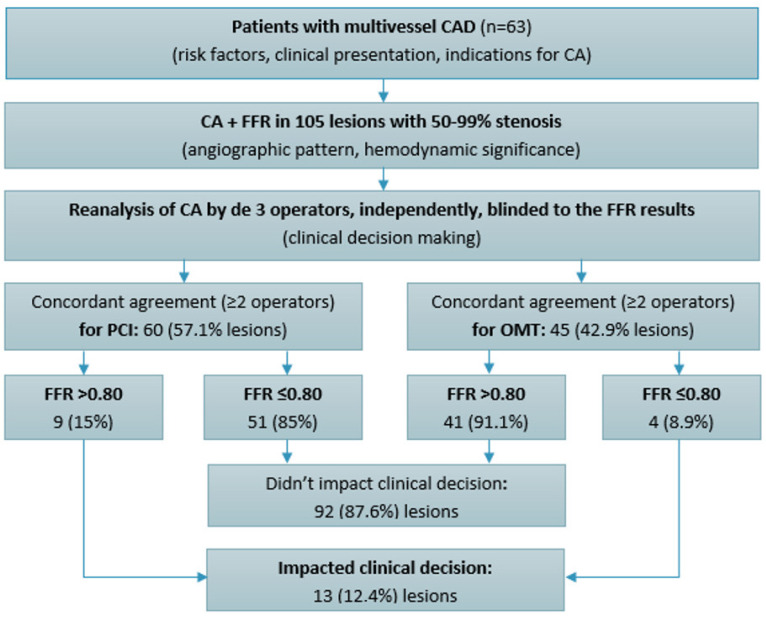
Study flowchart. CAD—coronary artery disease; CA—coronary angiography; FFR—fractional flow reserve; PCI—percutaneous coronary intervention; OMT—optimal medical therapy.

This was a retrospective observational study that included 63 patients with CAD assessed by CA, who underwent, at the same time, a functional evaluation by FFR of 105 lesions (50–99% artery lumen stenosis) in 2023 at the Institute of Cardiology, Chisinau, Republic of Moldova. Patients were assessed for age, sex, traditional risk factors, clinical status at presentation (stable angina, unstable angina, or NSTEMI), and clinical and paraclinical data serving as indications for CA (anginal symptoms, dyspnea, exercise test, prior myocardial infarction, and low left ventricular ejection fraction). CA was performed using GE Innova IGS angiography machines, following the standard protocol. The following angiographic characteristics of CAD were assessed: the number of diseased vessels and the location and severity of stenoses. Thus, the inclusion criteria for the study group were patients with CAD, defined as 50–99% lumen narrowing, as assessed by CA, which were subsequently evaluated by FFR measurement (Figure 1). Patients with STEMI and left main CAD and those with chronic total occlusions were excluded from this study.

### 2.2. Fractional Flow Reserve

**Figure 2:**
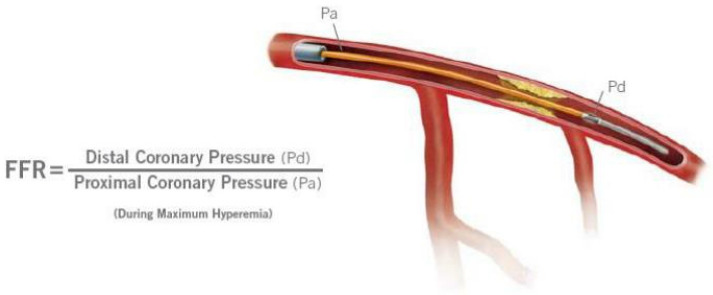
Fractional flow reserve assessment.

FFR assessment was performed for all 50–99% stenoses assessed by the primary operator at the time of CA. After an intravenous administration of heparin (70 IU/kg), a 0.014” intracoronary pressure wire, 175 cm (PressureWire X, Abbott Medical, MN, USA), was advanced distally to the stenosis (Figure 2). After an intracoronary administration of 100–200 mcg of isosorbide dinitrate (according to hemodynamics), reactive hyperemia was induced by intravenous adenosine (140–180 µg/kg/min) administration [15]. An FFR value of ≤0.80 was used as the cutoff to indicate the functional significance of CAD [16]. Percutaneous coronary intervention (PCI) was performed for lesions with an FFR ≤ 0.80, while lesions with an FFR > 0.80 were managed with optimal medical therapy (OMT), in accordance with current guideline recommendations [3]. If a patient had two or more functionally significant lesions in two or more arteries, staged PCI was preferred, with an interval of 2–4 weeks between the initial procedure and subsequent intervention. PCI was performed using standard percutaneous techniques, either via a radial, brachial, or femoral approach.

### 2.3. Visual Assessment Variability

Each CA was reanalyzed by three experienced interventional cardiologists (with a volume of >300 PCIs performed annually), independently of each other, to determine the angiographic severity of lesions and the need for revascularization based on the visual assessment and clinical presentation, and they were blinded to FFR values. Concordant agreement was considered when at least two interventional cardiologists made the same decision—to treat or not treat a lesion. Complete agreement, when all three interventional cardiologists had the same treatment decision, was also assessed. These results were matched with the FFR results to reveal where they had an impact to change clinical decision making.

### 2.4. Statistical Analysis

Statistical analysis was conducted using the Statistical Package for Social Sciences (SPSS), version 16. Categorical variables were reported as frequencies and percentages, while continuous variables were presented as the mean ± standard deviation. The normality of data distribution was assessed using the Kolmogorov–Smirnov test.

## 3. Results

### 3.1. Study Population

The mean age of the study population of 63 patients was 63,55 years (range 34 to 78 years), with 48 (76.2%) males and 15 (23.8%) females (Table 1). The most prevalent risk factors in this study group were as follows: hypertension—60 patients (95.2%), dyslipidemia—54 patients (85.7%), type 2 diabetes mellitus—40 patients (63.5%), a family history of cardiovascular disease—11 patients (17.5%), and smoking—9 patients (14.3%). In total, 105 lesions of 50–99% stenosis in 63 patients were identified, which were evaluated by FFR, averaging 1.67 lesions per patient.

Clinical presentation was diverse, including stable angina in 39 patients (61.9%), unstable angina in 20 patients (31.7%), and NSTEMI in 4 patients (6.4%). The indications for CA were predominantly the presence of typical angina pectoris (34 patients (53.9%)) and prior myocardial infarction (16 patients (25.4%)). Most patients—56 (88.9%)—who underwent CA did not have exercise testing within the 90-day pre-procedural period, and only 7 (11.1%) of them had a positive exercise test for documenting ischemia. In 92% of cases, coronary angiography was performed via radial access, which implies minimal local complications at the puncture site.

### 3.2. Lesion Characteristics

**Figure 3:**
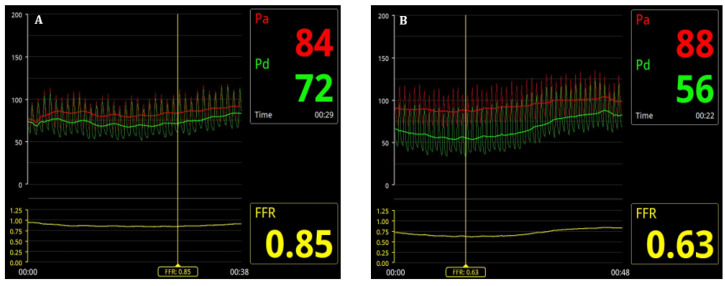
FFR measurement = Pd (distal pressure)/Pa (aortic pressure), during maximal hyperemia. Example of (**A**) non-significant FFR (>0.8) and (**B**) significant FFR (≤0.8).

In terms of angiographic characteristics, 33 (52.4%) patients in the study group had multivessel CAD (two- or three-vessel disease), and the majority of lesions were located in the left anterior descending artery (LAD)—53 (50.5%)—followed by lesions in the right coronary artery (RCA) and left circumflex artery (LCx) (Table 2). The primary operator visually assessed 58 (55.2%) of lesions as being severe and 47 (44.8%) of lesions as being moderate. Non-significant FFR values (>0.8) were found in 50 of the lesions (47.6%), which did not undergo PCI, while significant FFR values (≤0.8) were found in 55 of the lesions (52.4%), which underwent PCI (Figure 3). It is interesting to mention that 21 of the hemodynamic significant lesions (38.2%) were found in patients presenting with acute coronary syndrome (unstable angina or NSTEMI). This suggests a heightened need for an FFR assessment in ACS patients, where angiography may not adequately reflect the true functional severity of the lesions. The incorporation of FFR in these cases can help prevent unnecessary PCI or missed opportunities for revascularization.

Lesions located in the LAD showed hemodynamic significance (FFR ≤ 0.8) in 25 (47.2%) cases, those in the RCA in 15 (53.6%) cases, and those in the LCx in 15 (62.5%) cases. Moderate stenoses (50–74%) were hemodynamic significant (FFR ≤ 0.8) in 8 (17.1%) cases, and the majority of severe stenoses (75–99%) were hemodynamically significant in 47 (81.0%) cases.

### 3.3. Tailored Insights Based on Subgroup Analyses

Patients with multivessel disease were more likely to have functionally significant lesions compared to those with single-vessel disease. Additionally, individuals with risk factors like diabetes and hypertension had a higher prevalence of significant lesions, particularly in the LAD, which underscored the importance of a tailored revascularization strategy in these high-risk subgroups. The integration of FFR in patients with ACS or multivessel disease can optimize treatment decisions, reducing the potential for both over- and under-treatment.

### 3.4. Clinical Decision Making

**Figure 4:**
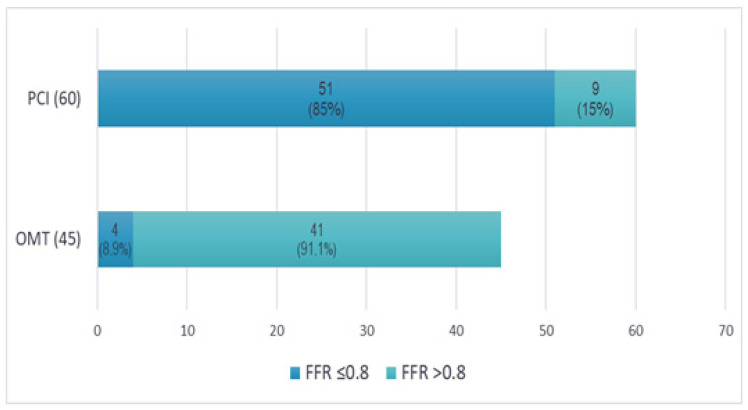
Discrepancy of clinical decisions with FFR assessment. Concordant agreement (≥2 out of 3 interventional cardiologists) to perform PCI for 60 lesions based on the angiographic appearance, while 9 (15%) of these had an FFR > 0.8. Conversely, a concordant agreement to defer PCI in favor of OMT was reached for 45 lesions, while 4 (8.9%) of these had an FFR ≤ 0.8.

Upon re-evaluating the angiographic images, three experienced interventional cardiologists (each performing over 300 PCIs annually) independently reviewed the cases, blinded to the FFR results. Concordant agreement (at least two out of three cardiologists making the same decision) was reached to proceed with PCI for 60 lesions (57.1%) based on an angiographic assessment and clinical presentation (Figure 4). Interestingly, nine of these lesions (15%) had FFR values that were functionally insignificant. Conversely, there was concordant agreement to defer PCI in favor of OMT for 45 lesions (42.9%). However, four of these lesions (8.9%) had FFR values that were functionally significant and required PCI. As a result, FFR had an impact to change the revascularization decision in 13 lesions (12.4%), both indicating PCI for 4 functionally significant lesions and deferring 9 functionally non-significant lesions for OMT. Notably, complete agreement (all three cardiologists making the same decision to either perform PCI or defer to OMT) was achieved in 82 lesions (78.1%). This result is consistent with other research on FFR [17] and quantifies the impact of FFR on therapeutic decisions, underscoring the ongoing gap in clinical practice between functional and visual assessments.

### 3.5. Clinical Guidelines Based on Findings

Based on this study’s findings, we offer the following clinical guidelines to help practitioners integrate FFR into their daily work:

1. Use FFR for Ambiguous Lesions: For angiographic lesions with moderate stenosis (50–74%), FFR should be routinely employed to assess functional significance. In our study, only 17.1% of moderate stenoses were found to be hemodynamically significant (FFR ≤ 0.8), underscoring the potential for FFR to refine treatment decisions.
2. Integrate FFR in ACS Patients: In patients presenting with acute coronary syndromes (unstable angina or NSTEMI), where visual assessments may be less reliable, FFR is crucial in guiding revascularization. As our findings show, 38.2% of functionally significant lesions were in ACS patients, making FFR particularly useful in this clinical context.
3. Address Inter-Observer Variability: In centers where multiple operators are involved in decision making, FFR should be used to resolve discrepancies in visual assessments. The high rate of discordant opinions (21.9% of cases) in our study suggests that a visual assessment alone may not be sufficient for accurate decision making.
4. FFR for Cost-Effective Care: Given the cost of PCI and the potential for unnecessary interventions, FFR should be applied to prevent over-treatment. In our study, FFR prevented unnecessary PCI in 15% of cases, demonstrating its value in reducing healthcare costs while improving outcomes.

These guidelines can help practitioners make more evidence-based decisions, especially in scenarios where angiography and visual assessments alone may lead to inappropriate treatment strategies.

## 4. Discussion

The current study evaluates the impact of FFR on revascularization decisions for atherosclerotic lesions with 50–99% stenosis in a group of 63 patients with CAD. Consistent with previous studies [18,19], the majority of our cohort were men over 60 years old with traditional risk factors like hypertension and dyslipidemia. Most often, the indication for CA is driven by typical angina symptoms, and rarely have these patients undergone non-invasive stress testing to document inducible ischemia, as is common in real-world clinical practice. This aligns with findings from other studies indicating that multivessel disease, particularly involving the LAD, is predominant in patients undergoing CA [20]. In our study, more than half of these lesions were functionally significant (FFR ≤ 0.80) and required revascularization via PCI.

One of the key findings in our study is that 38.2% of hemodynamically significant lesions were found in patients presenting with acute coronary syndrome. This emphasizes the need for FFR in more acute settings, as reported in other studies [21,22,23], where angiography alone may not provide a reliable assessment of the true functional severity of lesions, particularly in ACS cases where ischemia needs to be accurately documented to guide revascularization.

Despite the proven benefits of FFR, less than 10% of coronary procedures utilize intracoronary imaging or FFR to guide patient management, as reported by other investigators [24,25]. This is due to various practical limitations related to FFR measurement, such as the additional time and costs associated with using a coronary guidewire equipped with a distal pressure sensor and the need for intracoronary or intravenous adenosine administration [26]. Similar to the findings of Tonino et al. [27], our study shows that PCI might be occasionally performed on lesions that are functionally insignificant (FFR > 0.80), which could potentially be avoided if FFR measurements were routinely employed. For example, in our cohort, FFR identified functionally non-significant lesions in 15% of cases where PCI was initially planned based on an angiographic assessment, thus preventing unnecessary revascularization. This result also parallels the findings of Götberg et al. [28], who reported that FFR-guided strategies significantly reduce the number of stents used and overall procedural costs.

Our results also highlight the limitations of visual assessments, especially in intermediate stenoses. Consistent with previous studies [29], we found that 17% of lesions with moderate stenosis (50–74%) were hemodynamically significant, while 19% of severe stenoses (75–99%) were functionally insignificant, allowing the FFR-guided strategy to prevent unnecessary revascularization in these cases. The difference in visual and hemodynamic guidance has been found in other studies, where it reduced the need for stenting and flattened the added cost of the pressure wire [30,31]. These discrepancies between angiographic and functional significance are well documented and emphasize the added value of FFR in guiding appropriate treatment decisions. Our findings are in line with research by Curzen et al., which also reported that FFR could alter clinical decision making in a substantial number of cases [32].

In the present study, there was a concordant agreement among the operators (≥two out of three) to revascularize 57.1% of the lesions based on the angiographic appearance, while 15% of these were functionally non-significant according to the FFR measurements. On the other hand, a concordant agreement to defer PCI in favor of OMT was reached for 42.9% of the lesions, while 8.9% of these were functionally significant. Our findings are comparable to those from previous studies [17] that have demonstrated similar discrepancies, further underscoring the importance of incorporating FFR into routine clinical practice to improve decision making. This study adds a new layer of insight into the variability between experienced interventional cardiologists. In 21.9% of cases, the cardiologists did not reach a unanimous decision regarding treatment. This variability in clinical decision making based on an angiographic assessment alone has been previously reported, but our study extends the understanding of its magnitude in a real-world setting where FFR is introduced into the process.

Despite its well-established clinical value, the routine use of FFR in daily practice is not consistently cost-effective [30], making it important to identify the group of lesions where FFR can yield truly significant results. The clinical and cost implications of such a shift in the decision-making process, especially in resource-limited areas like the Republic of Moldova, require further evaluation in larger studies. In reality, many factors can influence the physician’s decision to perform revascularization, including patient preference, treatment compliance, lesion complexity, the feasibility of achieving complete revascularization, and additional costs, all of which can lead to a decision that may be discordant with the FFR outcome.

## 5. Conclusions

Angiographically guided decision making on the revascularization of CAD is largely consistent with the FFR results. However, in 12.4% of cases, FFR led to a change in treatment strategy, both indicating PCI for hemodynamically significant lesions and deferring PCI for non-significant ones. These findings highlight the importance of incorporating FFR into routine clinical practice to improve the precision of revascularization decisions. In the future, a broader adoption of FFR could reduce unnecessary interventions, enhance patient outcomes, and optimize healthcare resources.

## Figures and Tables

**Table 1:** Demographic and clinical characteristics of the study population.

Patient Characteristics		N = 63 (100%)
Mean age (years ± standard deviation) 63.55 ± 8.7		63.55 ± 8.7
*Sex*	Male	48 (76.2%)
	Female	15 (23.8%)
*Risk* *Factors*	Smoking	9 (14.3%)
	Type 2 Diabetes Mellitus	40 (63.5%)
	Hypertension	60 (95.2%)
	Dyslipidemia	54 (85.7%)
	Family History	11 (15.5%)
*Clinical* *Presentation*	Stable Angina	39 (61.9%)
	Unstable Angina	20 (31.7%)
	NSTEMI	4 (6.4%)
*Indications* *for CA*	Typical Angina	34 (53.9%)
	Dyspnea	12 (19%)
	Atypical Presentation	5 (7.9%)
	Positive Stress Test	7 (11.1%)
	Prior Myocardial Infarction	16 (25.4%)
	Left Ventricular Systolic Dysfunction (EF < 50%)	13 (20.6%)
*Stress* *Testing*	No Pre-Procedural Stress Testing	56 (71.4%)
	Positive Stress Test	7 (11.1%)
*Puncture* *Site*	Radial Access	58 (92%)
	Femoral Access	4 (6.4%)
	Brachial Access	1 (1.6%)
*Diseased* *Vessels*	One-Vessel disease	30 (47.6%)
	Two-Vessel disease	23 (36.5%)
	Three-Vessel disease	10 (15.9%)

**Table 2:** Angiographic characteristics and hemodynamic significance of the lesions.

*Angiographic Characteristics* *105 (100%) Lesions*			Hemodynamic Significance	
			FFR > 0.8 50 (47.6%) Lesions	FFR ≤ 0.8 55 (52.4%) Lesions
*Lesion* *Location*	LAD	53 (50.5%)	28 (52.8%)	25 (47.2%)
	RCA	28 (44.4%)	13 (46.4%)	15 (53.6%)
	LCx	24 (38.1%)	9 (37.5%)	15 (62.5%)
*Stenosis Severity*	Moderate (50–74%)	47 (44.8%)	39 (83.0%)	8 (17.0%)
	Severe (75–99%)	58 (55.2%)	11 (19.0%)	47 (81.0%)

## Data Availability

The data presented in this study are available on request from the corresponding author. The data are not publicly available due to privacy and ethical restrictions.
